# Activation of Notch-1 signaling pathway in macrophages to secrete PD-L1 and regulate cytotoxicity of CAR-T cells in diffuse large B-cell lymphoma

**DOI:** 10.18632/aging.205463

**Published:** 2024-01-22

**Authors:** Weijing Li, Lili Wu, Chen Huang, Hongqing Ma, Lianjing Wang, Wei Liu, Lihong Liu

**Affiliations:** 1 Department of Hematology, The Fourth Hospital of Hebei Medical University, Shijiazhuang, China; 2 Department of General Surgery, The Fourth Hospital of Hebei Medical University, Shijiazhuang, China

**Keywords:** diffuse large B-cell lymphoma, macrophages, Notch-1/IRE1/XBP1s signaling pathway, PD-L1, CAR-T cells

## Abstract

Objective: To investigate the mechanism of action of the Notch-1/IRE1/XBP1s signaling pathway in diffuse large B-cell lymphoma (DLBCL).

Methods: The expressions of relevant proteins were detected by Western blotting. The effect of myeloid-specific knockout of Notch-1 on lymphoma progression was observed by mouse tumor transplantation and imaging. The apoptosis of chimeric antigen receptor T-cell therapy (CAR-T) cells were detected by flow cytometry, and the proliferation of CAR-T cells was detected by wound healing assay and cell counting kit-8 (CCK8) assay.

Results: Lymphoma cells mediated the Notch-1 signaling pathway in bone marrow-derived macrophages and promoted the activation of STAT3 and STAT6 in bone marrow-derived macrophages. Myeloid-specific knockout of Notch-1 could inhibit the progression of lymphoma. Lymphoma cells enhanced the expression of p-PERK, p-IRE1α, ATF6, IL-6, IL-4, p-AKT, CD9, CD63 and PD-L1 in bone marrow-derived macrophages by mediating the Notch-1 signaling pathway. Knockout of Notch-1 in macrophages alleviated, to some extent, the suppression of killing activity of CAR-T cells, while activation of Notch-1 in macrophages inhibited proliferation and promoted apoptosis of CAR-T cells. The PD-L1 antibody significantly restored the cytotoxicity and proliferation of CAR-T cells, and inhibited their apoptosis.

Conclusion: Activation of the Notch-1/IRE1/XBP1s signaling pathway in myeloid macrophages promotes the secretion of IL-6 and IL-4 as well as PD-L1, thereby inhibiting the activity and proliferation of CAR-T cells and promoting their apoptosis.

## INTRODUCTION

Diffuse large B-cell lymphoma (DLBCL) is a malignant tumor originating from B lymphocytes, accounting for 30–40% of non-Hodgkin's lymphoma (NHL), and is one of the most common subtypes of lymphoma [1]. In recent years, the global incidence of DLBCL has increased year by year, posing a serious threat to human health [2]. Studies have shown that the dysregulation of cytokines, immune cells and signaling pathways in the tumor microenvironment plays a decisive role in the occurrence and development of DLBCL [3]. The suppression of the programmed death-1 (PD-1)/programmed death-ligand 1 (PD-L1) pathway has achieved some progress in improving patient survival in immunotherapy, but it still has some limitations [4]. Chimeric antigen receptor T-cell therapy (CAR-T) has displayed significant efficacy in some DLBCL patients with drug resistance, but its efficacy is not durable [5]. Therefore, in-depth research of regulatory mechanisms of signaling pathways and cytokines in the tumor immune microenvironment is of great significance for overcoming drug resistance in DLBCL patients and improving the efficacy of new therapeutic strategies.

In the tumor microenvironment, immune cells are crucial to the occurrence and development of tumors. The tumor microenvironment is a complex biological system, including tumor cells, immune cells, extracellular matrix and supporting cells. Immune cells, such as T cells, macrophages, and dendritic cells, can recognize and eliminate tumor cells during immune surveillance. Under certain circumstances, however, tumor cells can evade immune surveillance, deactivating immune cells through immunosuppressive mechanisms, and leading to the development and progression of tumors. In particular, mononuclear macrophages, which are key immune cells, have a significant impact on tumor formation, growth, invasion and migration [6]. Macrophages are a group of bone marrow-derived cells with multiple functions, which play important roles in both normal physiological and pathological activities. As a key component of the immune system, bone marrow-derived macrophages can regulate inflammatory responses, phagocytize pathogenic microorganisms, remove dead cells, and promote tissue repair, occupying an important position in immune regulation including anti-tumor and anti-infection immune responses. It has turned out that the endoplasmic reticulum stress (ERS) plays a pivotal role in regulating macrophage function and tumor microenvironment [7]. The ERS pathway can regulate the biological functions of macrophages by activating different signaling pathways, such as the IRE1/XBP1s signaling pathway [8].

Notch is a highly conserved cell-cell communication pathway, which exists in a variety of organisms, including Drosophila, nematodes and vertebrates. The Notch signaling pathway includes four receptors (Notch-1-4) [9] and five ligands (Delta-like 1, 3, 4 and Jagged 1, 2) [10]. As a member of the Notch signaling pathway, Notch-1 is of great importance in the development of tumors [11, 12]. Exosomes are one subtype of secreted vesicles that encapsulate and deliver bioactive molecules for cell-cell communication. In recent years, the role of exosomes in tumorigenesis, tumor progression and metastasis has been widely studied. It has been found that exosomes produced by tumor cells are rich in various immunomodulatory molecules, such as PD-L1, which can enhance tumor immune escape. According to recent studies, the AKT signaling pathway is closely related to the biogenesis and function of exosomes. Activation of AKT can increase the production of exosomes in tumor cells, which in turn affects the biological behavior of adjacent and distant cells [13–15]. PD-L1 fulfills an important function in tumor immune escape. The close association between AKT signaling pathway and PD-L1 expression has been verified, i.e., AKT activation promotes PD-L1 secretion by exosomes. These exosomes, which are rich in PD-L1 and inhibit immune cell activity, further contribute to tumor immune escape. CAR-T cell therapy is a revolutionary cancer treatment that genetically engineers the patient's own T cells to recognize and attack cancer cells. CAR-T cell therapy has been demonstrated to be effective in the treatment of some hematologic tumors, such as acute lymphocyte leukemia and DLBCL [16, 17]. Therefore, the effects of the Notch-1/IRE1/XBP1s signaling pathway and PD-L1 secretion on the activity, proliferation and apoptosis of CAR-T cells were explored in this study.

## MATERIALS AND METHODS

### Animal models

A total of 18 SPF male BALB/c-nu mice aged about 5 weeks and weighing 16–20 g were purchased from SPF (Beijing) Biotechnology Co., Ltd. They were housed in an SPF animal house with a covered and medium-efficiency air filter on a laminar flow cabinet under constant temperature (20°C–26°C) and constant humidity (50–60%), and fed with sterile standard pellet feed. Materials in contact with mice were autoclave sterilized. The procedures for animal handling and testing were approved by the Institutional Animal Care and Use Committee of The Fourth Hospital of Hebei Medical University, and conformed to the Guide for the Care and Use of Laboratory Animals of the National Institutes of Health.

### Cell lines

Raji-Luc-GFP cells purchased from BMCR were cultured with complete medium containing 90% RPMI-1640 medium (C22400500BT, Gibco, USA), 10% fetal bovine serum (FBS) and 1% penicillin-streptomycin in a 5% CO_2_ incubator at 37°C and saturated humidity. The cells were passaged every 2–3 days, and those of the 3rd-5th generations in the logarithmic growth phase were then collected for later experiments. Mouse and humanized CAR-T cells were purchased from Hebei Senlang Biotechnology Co., Ltd. (Hebei, China). The ratio of CD4+/CD8+ in CAR-T cells is 1:1, and the target of CAR-T cells is CD19.

### Construction of mice with myeloid-specific knockout of Notch-1

Mice with myeloid-specific knockout of Notch-1 were generated from floxed Notch-1 (Notch-1FL/FL) mice using the lysozyme 2 (Lyz2) promoter. First, homozygous loxP flanking Notch-1 mice were mated with homozygous Lyz2-Cre mice to generate F1 mice that were heterozygous for the loxP flanking Notch-1 allele and heterozygous for Lyz2-Cre. Then these F1 mice were backcrossed with homozygous loxP flanking Notch-1 mice, resulting in Notch-1M-KO offspring (25%) homozygous for the loxP flanking Notch-1 allele and heterozygous for the Lyz2-Cre allele.

### Extraction and differentiation of primary mouse bone marrow cells

After cervical dislocation of the mice aged 6–10 weeks, the femur and tibia were taken out, and the muscle tissues around the bone were removed as far as possible with scissors and tweezers to avoid damage to the bone. Then the bone was washed twice with sterile PBS, and cut off at both ends with scissors. The bone marrow cavity was repeatedly washed using a syringe with PBS until the bone marrow was completely discharged and the bone became white. The bone marrow suspension was collected and filtered through a 200-mesh nylon strainer to remove debris and muscle tissues. The filtrate was centrifuged at 1200 rpm for 5 minutes and the supernatant was discarded. The red blood cells were lysed with 2 mL of 1-fold ammonium chloride solution and the cells were incubated at room temperature for 5 minutes. After 10 mL of balanced salt solution was injected, the cells were centrifuged at 1200 rpm for 5 minutes, and the supernatant was discarded. Then the cells were washed once with cold PBS and resuspended in prepared culture medium to harvest the mouse bone marrow cells. The mouse bone marrow cells were resuspended in RPMI-1640 medium with 10% FBS, counted and seeded at a density of 0.5–1 × 10^6^/mL. Then 1 mL of cell suspension and GM-CSF (20 mg/mL) were added to each well of a 24-well plate on Day 0, with 3/4 of the volume changed and cytokines added every 2 days. On Day 5–8, the plate was gently tapped to collect suspension cells and centrifuged at 1200 rpm for 5 minutes, and the supernatant was discarded. The cells were resuspended in the culture medium, the cell concentration was adjusted to 1 × 10^6^/mL, and GM-CSF (20 ng/mL) was added. Then the cells were cultured in 100 mm dishes or 6-well plates in a 5% CO_2_ incubator for 1–2 days at 37°C. Finally, the suspension cells were collected as more mature bone marrow-derived macrophages.

### Co-culture of bone marrow-derived macrophages with mouse lymphoma A20 cells

Bone marrow-derived macrophages were seeded into Transwell chambers and cultured for 24 hours. Mouse lymphoma A20 cells were seeded into culture plates and cultured for 24 hours. The culture medium was added with IXA4 (40 mg/mL) and replaced the next day. The Transwell chamber was placed in the culture plate, and 24 hours later, the chamber and culture medium were removed. The cells were washed twice with PBS and fixed with 4% paraformaldehyde.

### Co-culture of bone marrow-derived macrophages and mouse lymphoma A20 cells with CAR-T cells

CAR-T cells were cultured in Transwell chambers for 24 hours. Bone marrow-derived macrophages and A20 cells were co-cultured for 24 hours, and the medium was added with IXA4 (40 mg/mL). The Transwell chamber was placed in the culture plate and the culture medium was added. 24 hours later, the chamber was removed and the culture medium was discarded. The cells were washed twice with PBS and fixed with 4% paraformaldehyde.

### Isolation of exosomes

FBS was centrifuged at 10,000 g overnight and then filtered through a 0.2 μm syringe filter (Millipore, Burlington, MA, USA). The exosome-deficient FBS was used for cell culture (DMEM supplemented with 10% exosome-free FBS). To isolate exosomes, the cell supernatant was collected and centrifuged at 2000 g and 10,000 g for 30 minutes at 4°C. The final supernatant was filtered through a 22 μm syringe filter (Millipore, Burlington, MA, USA) and centrifuged at 120,000 g for 70 minutes. Afterwards, the spheroidized vesicles were washed with PBS and centrifuged again at 120,000 g for 70 minutes. Finally, the extracellular markers were detected by Western blotting to validate the result.

### Mouse tumor transplantation and imaging

Raji-Luc-GFP cells in the logarithmic growth phase were harvested, washed twice with PBS, and suspended in serum-free RPMI-1640 medium. After the density was adjusted to 5 × 10^4^ cells per 0.1 mL of medium, the tumor cell suspension was carefully injected through the tail vein into mice (5 × 10^5^ cells/mouse). During the experiment, the state of the mice was observed every day. On Day 3 after injection, tumor components showed by biopsy demonstrated the success of modeling. Then the growth of tumors in mice was examined every 7 days by *in vivo* small animal imaging, and the survival status of mice was observed every day.

### Western blotting

The cells were mixed with 490 μL of RIPA lysate, 5 μL of protease inhibitor PMSF, and 5 μL of phosphatase inhibitor and placed on ice for later use. The proteins were extracted, and their concentration was quantified by the BCA assay. Afterwards, separation and concentration gels were prepared. The proteins were diluted with 5× SDS-PAGE loading buffer, boiled at 100°C for 5 minutes, and placed on ice for later use. Then 30 mg of proteins were loaded, subjected to electrophoresis, and transferred onto a PVDF membrane. The membrane was blocked with TBST blocking buffer containing 5% skim milk powder for 2 hours at room temperature on a shaking table, and incubated with diluted primary antibodies (1:1000) against Notch-1 (ab52627, Abcam, UK), p-STAT3 (ab267373, Abcam), t-STAT3 (ab109085, Abcam), p-STAT6 (ab263947, Abcam), t-STAT6 (ab32108, Abcam), p-PERK (3179, Cell Signaling, USA), p-IRE1α (ab124945, Abcam), ATF6 (ab227830, Abcam), IL-6 (ab233706, Abcam), IL-4 (ab34277, Abcam), p-AKT (ab8933, Abcam), t-AKT (ab300743, Abcam), CD9 (ab236630, Abcam), CD63 (ab134045, Abcam), PD-L1 (ab213480, Abcam) and GAPDH (ab2293, Abcam) at room temperature for 1 hour, and then placed in a refrigerator at 4°C. After 24 hours, the PVDF membrane was taken out and washed three times with 1× TBST on a shaking table (10 min/time), followed by incubation with corresponding secondary antibodies (1:2000) at room temperature for 2 hours. After washing three times with TBST (10 min/time), the PVDF membrane was immersed in the luminescent solution, and detected using the software.

### Cell counting kit-8 (CCK8) assay

The CAR-T cells in the logarithmic growth phase were digested, diluted and counted with a cell counting plate. At 24 h, 48 h and 72 h, the medium was replaced and 10 μL of CCK8 buffer was added to each well. After culture away from light for 2 hours, the cell viability was detected at 450 nm. The assay was repeated three times and the data were recorded.

### Wound healing assay

The CAR-T cells in good growth status were harvested. Upon reaching 90% confluence, they were digested routinely and centrifuged at 1200 rpm for 5 minutes. The cell precipitate was resuspended, the cells were then counted, and the cell concentration was adjusted to 1.0 × 10^5^, with three replicates in each dish. 200 μL of cell suspension was collected, inoculated into a petri dish, and added with culture medium until the total volume was 6 mL. Afterwards, the cells were evenly dispersed, and cultured in a 5% CO_2_ incubator at 37°C for 2 weeks. When there were more than 50 visible colonies, the culture was terminated and the medium was discarded, and the cells were washed three times with PBS. Then the cells were fixed with 5 mL of methanol each well for 15 minutes, and the fixative was discarded. After washing with PBS three times, the cells in each well were stained for 20 minutes, rinsed with tap water, and naturally air-dried. Finally, they were photographed and counted using ImageJ software.

### Flow cytometry of CAR-T cells

An appropriate number of CAR-T cells in the logarithmic growth phase were collected, inoculated into a 6-well plate and cultured until reaching 90% confluence. After washing three times with pre-cooled PBS, the cells were gently mixed with 100 μL of 1× binding buffer, and stained with Annexin V or PI away from light at room temperature. After 15 min, 300 μL of 1× binding buffer was added, and the cell suspension was transferred to a 5 mL flow tube and detected within 1 hour using flow cytometry.

### Statistical analysis

SPSS 22.0 software was used for statistical analysis, and GraphPad Prism software 9.0 for plotting. The results were described by mean ± SD, and compared by *t*-test between the two groups. The assays were repeated at least three times in each group, and *P* < 0.05 was considered statistically significant.

## RESULTS

### Notch-1 knockout efficiency

**Figure 1:**
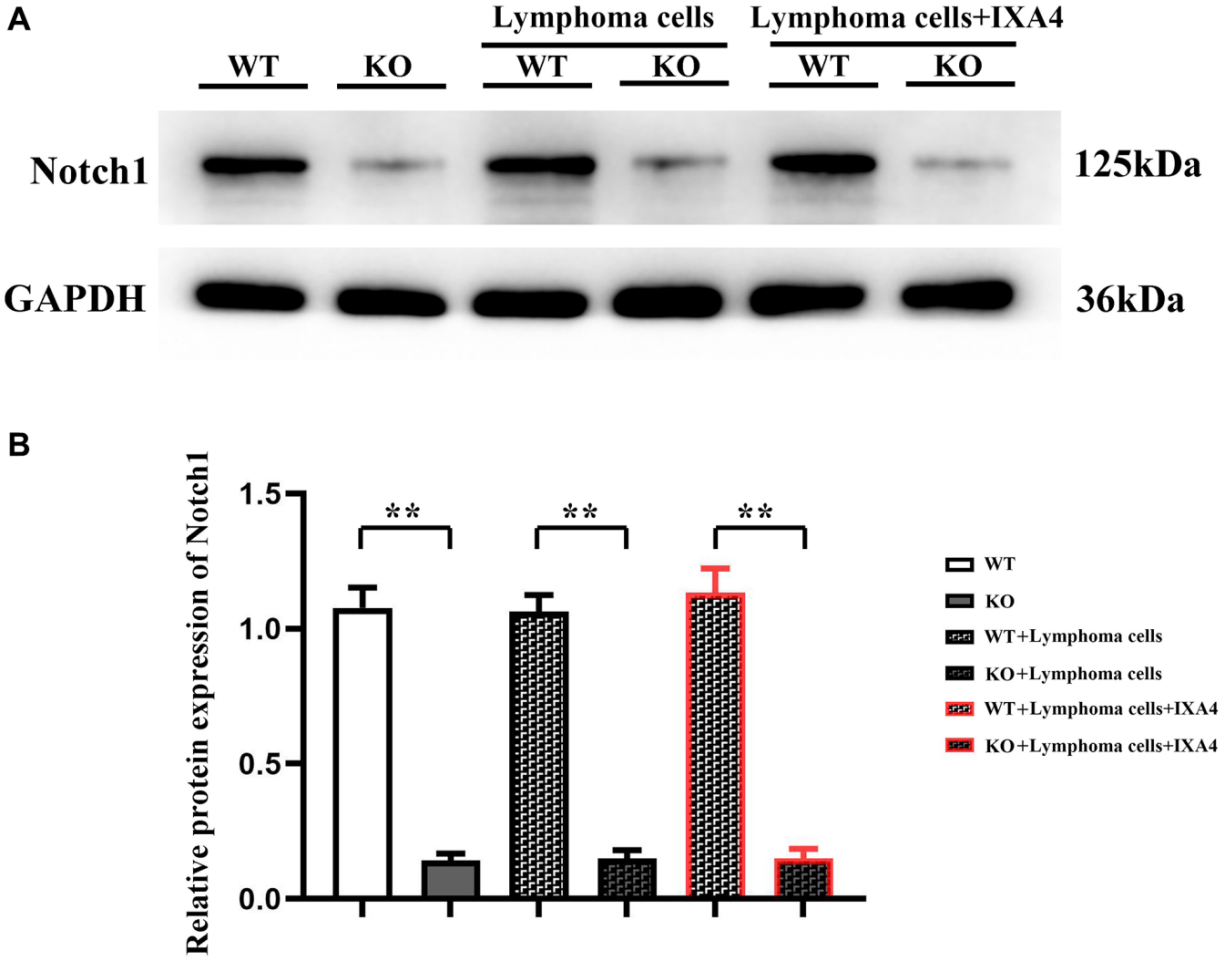
**Western blotting of Notch-1.** (**A**) Protein bands of Notch-1; (**B**) Relative protein expression of Notch-1. (WT group vs. KO group; WT + lymphoma cell group vs. KO + lymphoma cell group; WT + lymphoma cell + IXA4 group vs. KO + lymphoma cell + IXA4 group; ^**^P < 0.01, ^ns^*P* > 0.05; *N* = 3).

Notch-1 gene plays an important role in cell signal transduction, proliferation and differentiation. To explore the effect of Notch-1 in bone marrow-derived macrophages on DLBCL cells, Notch-1 conditional knockout mice were constructed. The bone marrow-derived macrophages were cultured, the expression level of Notch-1 protein was detected, and the Notch-1 knockout efficiency was determined in advance. Compared with WT group, KO group had a significantly decreased expression level of Notch-1 (*P* < 0.01). The results confirmed that Notch-1 conditional knockout mice were successfully constructed, with only trace amounts of Notch-1 expression observed, which could be used for later experiments (Figure 1).

### Effect of lymphoma cells on Notch-1 protein expression in bone marrow-derived macrophages

To investigate the effect of bone marrow-derived macrophages on lymphoma cells, whether lymphoma cells affect the expression level of Notch-1 in bone marrow-derived macrophages was first explored, and then lymphoma cells were co-cultured with bone marrow-derived macrophages. The results showed that compared with WT + lymphoma cell group, the expression level of Notch-1 had no significant difference in KO + lymphoma cell group and KO group (*P* > 0.05). It can be inferred that lymphoma cells do not affect the activation of the Notch-1 signaling pathway in bone marrow-derived macrophages.

Furthermore, IXA4, an activator of IRE1/XBP1s, was used to clarify whether the activation of IRE1/XBP1s has a regulatory effect on the Notch-1 expression. The results showed that the expression level of Notch-1 in KO + lymphoma cell + IXA4 group remained very low compared with WT + lymphoma cell + IXA4 group (*P* < 0.01), but was similar to that in KO group (Figure 1). The results suggested that the Notch-1 expression in the bone marrow-derived macrophages of Notch-1 conditional knockout mice is consistently suppressed and remains unchanged regardless of whether they are co-cultured with lymphoma cells and whether IXA4 is used. The findings lay a foundation for further exploration of Notch-1 and its molecular mechanisms.

### Lymphoma cells promoted activation of STAT3/STAT6 signaling pathway in bone marrow-derived macrophages

**Figure 2:**
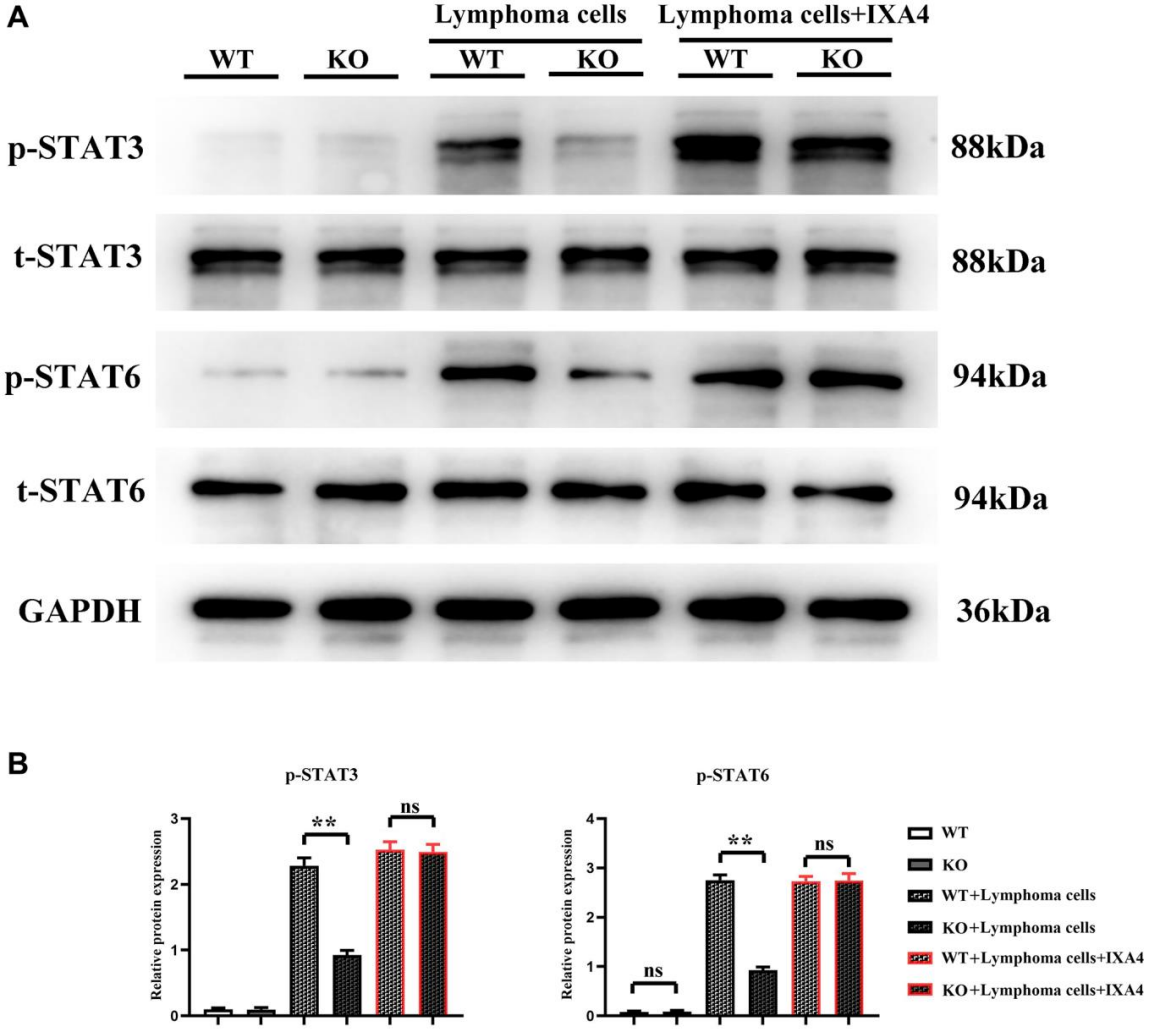
**Western blotting of p-STAT3 and p-STAT6.** (**A**) Protein bands of p-STAT3 and p-STAT6; (**B**) Relative protein expressions of p-STAT3 and p-STAT6. (WT group vs. KO group; WT + lymphoma cell group vs. KO + lymphoma cell group; WT + lymphoma cell + IXA4 group vs. KO + lymphoma cell + IXA4 group; ^**^P < 0.01, ^ns^*P* > 0.05; *N* = 3).

Whether lymphoma cells activate the STAT3/STAT6 signaling pathway in bone marrow-derived macrophages was investigated. It was found that the expression levels of t-STAT3 and t-STAT6 were not different between WT group and KO group, suggesting that the total protein levels of STAT3 and STAT6 are not upregulated at the transcriptional and translational levels after Notch-1 knockout. There was also no difference in the expression levels of p-STAT3 and p-STAT6 between the two groups, suggesting that Notch-1 knockout does not alter the activation levels of STAT3 and STAT6. Compared with WT group and KO group, KO + lymphoma cell group and WT + lymphoma cell group had significantly increased expression levels of STAT3 and STAT6, indicating that lymphoma cells can activate STAT3 and STAT6. The expression levels of p-STAT3 and p-STAT6 in KO + lymphoma cell group were significantly inhibited compared with WT + lymphoma cell group, and their levels only slightly increased compared with WT group and KO group. The results demonstrated that lymphoma cells can activate the Notch-1 downstream STAT3/STAT6 signaling pathway. Besides, IXA4 did not change the expressions of STAT3 and STAT6. In KO + lymphoma cell + IXA4 group, the expressions of activated STAT3 and STAT6 were similar to that in WT + lymphoma group, while the expressions of p-STAT3 and p-STAT6 were significantly higher than those in KO + lymphoma cell group (Figure 2). It can be seen that the activation of IRE1/XBP1s positively activates the STAT3/STAT6 signaling pathway.

### Lymphoma cells promoted activation of ERS signaling pathway in bone marrow-derived macrophages

**Figure 3:**
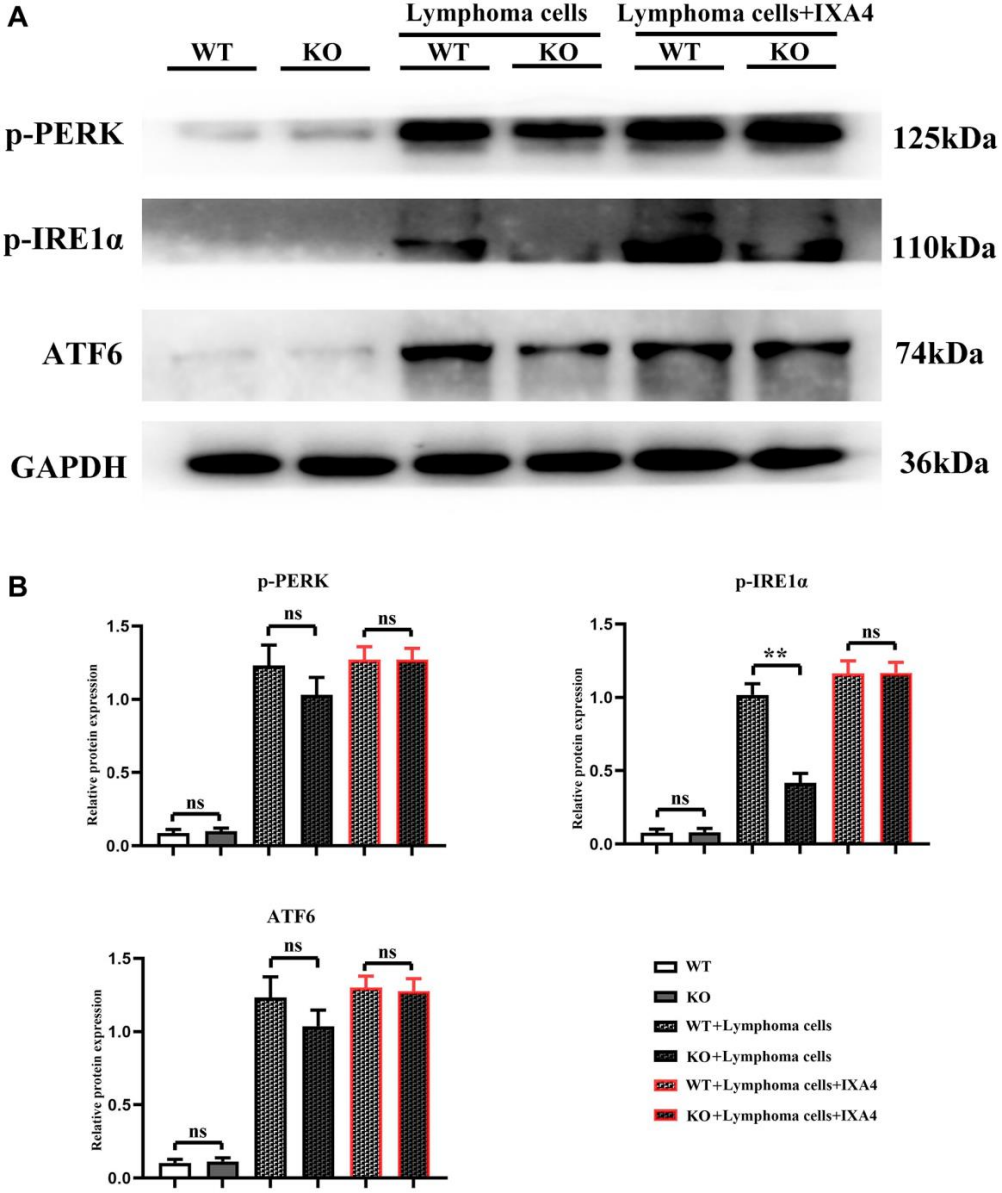
**Western blotting of p-PERK, p-IRE1α and ATF6.** (**A**) Protein bands of p-PERK, p-IRE1α and ATF6; (**B**) Relative protein expressions of p-PERK, p-IRE1α and ATF6. (WT group vs. KO group; WT + lymphoma cell group vs. KO + lymphoma cell group; WT + lymphoma cell + IXA4 group vs. KO + lymphoma cell + IXA4 group; ^**^P < 0.01, ^ns^*P* > 0.05; *N* = 3).

The results showed that in WT group and KO group, p-PERK, p-IRE1α and ATF6 were all lowly expressed, suggesting that the ERS level is low. Compared with WT group and KO group, the expression levels of p-PERK, p-IRE1α and ATF6 were significantly upregulated in WT + lymphoma cell group, indicating that lymphoma cells can simultaneously activate the three oxidative stress pathways. Compared with WT + lymphoma cell group, the expressions of p-PERK and ATF6 slightly decreased and the expression of p-IRE1α was significantly inhibited in KO + lymphoma cell group, proving that p-IRE1α is a key downstream gene of Notch-1, and Notch-1 knockout can significantly block the expression of p-IRE1α. Furthermore, IXA4 was used to activate IRE1α. The results showed that compared with KO + lymphoma cell group, the expression of p-IRE1α was dramatically upregulated in KO + lymphoma cell + IXA4 group, but there was no significant difference in the expression levels of p-PERK and ATF6 (Figure 3). To sum up, IXA4 can activate the IRE1α signaling pathway but has no impact on the other two ERS signaling pathways.

### Lymphoma cells activated Notch-1/IRE1α/IL-6 and IL-4 signaling pathways in bone marrow-derived macrophages

**Figure 4:**
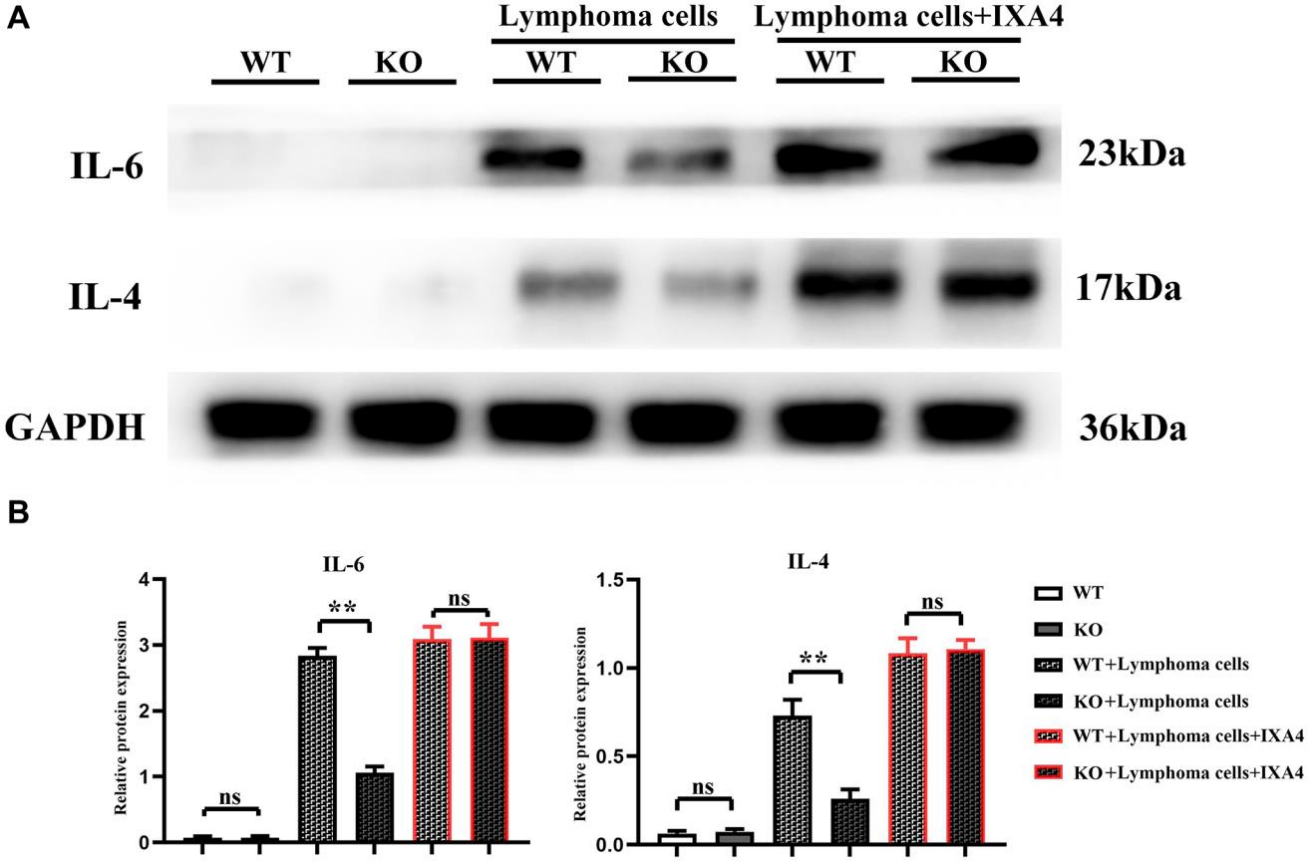
**Western blotting of IL-4 and IL-6.** (**A**) Protein bands of IL-4 and IL-6; (**B**) Relative protein expressions of IL-4 and IL-6. (^**^P < 0.01; *N* = 3).

The expression levels of IL-4 and IL-6 were low in WT group and KO group, but they significantly increased in WT + lymphoma cell group, suggesting that lymphoma cells can activate IL-4 and IL-6 in bone marrow-derived macrophages. The expressions of IL-4 and IL-6 in KO + lymphoma cell group were significantly inhibited compared with WT + lymphoma cell group, demonstrating that Notch-1 is a key upstream molecule regulating the expressions of IL-4 and IL-6. When IXA4 was used, the expressions of IL-4 and IL-6 were upregulated in WT + lymphoma cell + IXA4 group compared with WT + lymphoma cell group. It can be seen that lymphoma cells and IXA4 may interact with each other to promote the expressions of downstream IL-4 and IL-6 (Figure 4). At the same time, Notch-1, although knocked out, activating its downstream IRE1/XBP1s signaling pathway can also significantly upregulate the expressions of downstream IL-4 and IL-6.

### Lymphoma cells promoted activation of AKT signaling pathways and release of PD-L1-containing exosomes in bone marrow-derived macrophages

**Figure 5:**
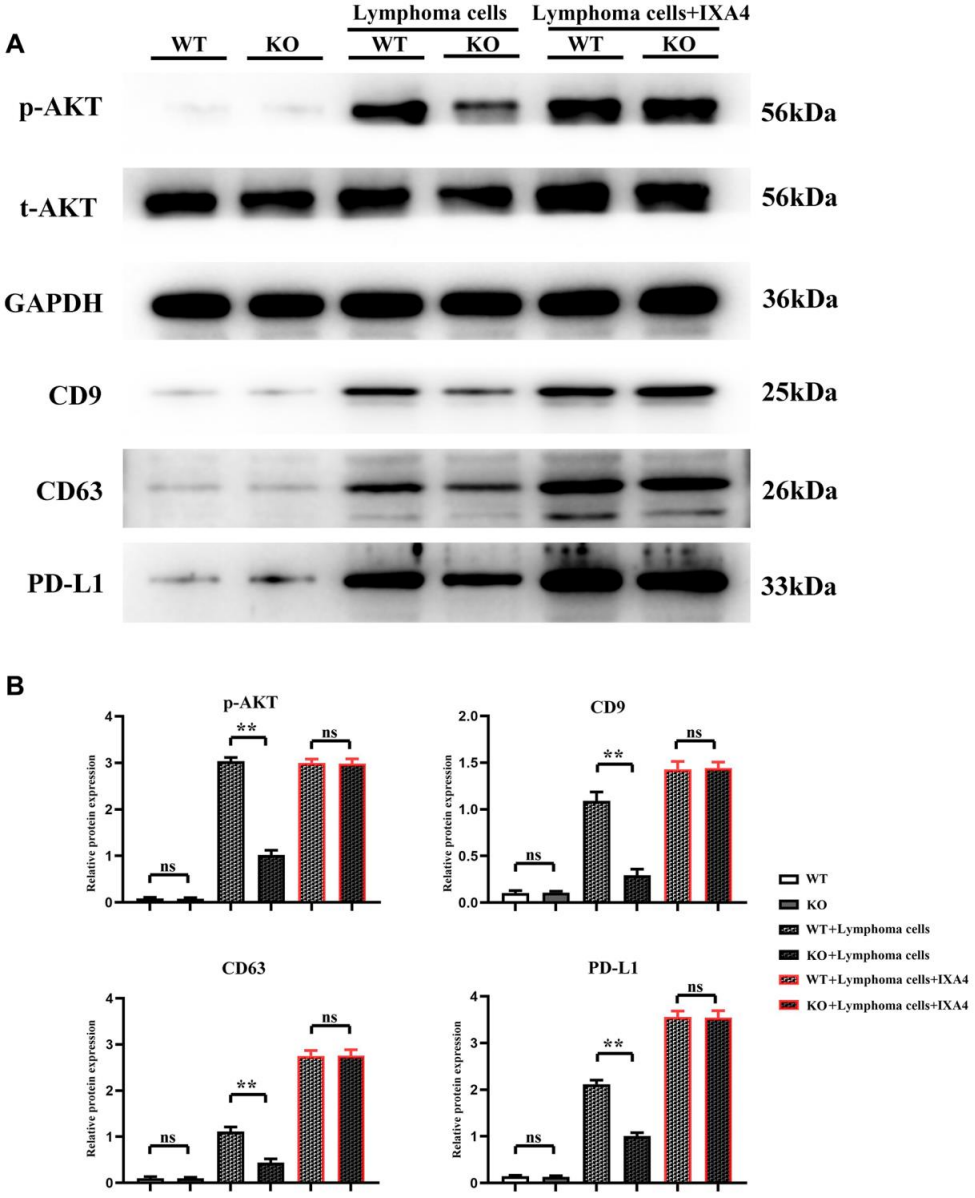
**Western blotting of AKT signaling pathway-related proteins, exosome-associated proteins and PD-L1.** (**A**) Protein bands of p-AKT, CD9, CD63 and PD-L1; (**B**) Relative protein expressions of p-AKT, CD9, CD63 and PD-L1. (WT + lymphoma cell + CAR-T group vs. KO + lymphoma cell + CAR-T group; WT + lymphoma cell + CAR-T + PD-L1 neutralizing antibody group vs. KO + lymphoma cell + CAR-T + PD-L1 neutralizing antibody group; ^**^P < 0.01, ^ns^*P* > 0.05; *N* = 3).

The expression level of p-AKT had no significant difference between WT group and KO group, indicating that Notch-1 knockout does not affect the expression and activation of downstream AKT. At the same time, the expression levels of CD9, CD63 and PD-L1 had no significant difference and remained lower between the two groups, suggesting that Notch-1 knockout does not affect the secretion of PD-L1 by macrophages. Compared with WT group, the expression levels of p-AKT, CD9, CD63 and PD-L1 significantly increased in WT + lymphoma group, suggesting that lymphoma cells can activate the AKT signaling pathway. The expression levels of p-AKT, CD9, CD63 and PD-L1 in KO + lymphoma group were significantly lower than those in WT + lymphoma group, demonstrating that Notch-1 is a key upstream molecule for the activation of AKT pathway in macrophages. Moreover, the expression levels of p-AKT, CD9, CD63 and PD-L1 in WT + lymphoma cell + IXA4 group showed little changes compared with WT + lymphoma cell group, further proving that activation of the IRE1/XBP1s signaling pathway by IXA4 does not activate the downstream AKT signaling pathway. The expression levels of p-AKT, CD9, CD63 and PD-L1 in KO + lymphoma cell + IXA4 group were significantly higher than those in KO + lymphoma cell group but similar to those in WT + lymphoma cell group and WT + lymphoma cell + IXA4 group, proving that IRE1/XBP1s is an important pathway downstream of Notch-1 (Figure 5). To sum up, IRE1/XBP1s is a major Notch-1 downstream pathway that regulates the secretion of PD-L1-containing exosomes, and its activation will significantly enhance the ability of macrophages to secrete PD-L1-containing exosomes.

### Myeloid-specific knockout of Notch-1 could inhibit the progression of lymphoma

**Figure 6:**
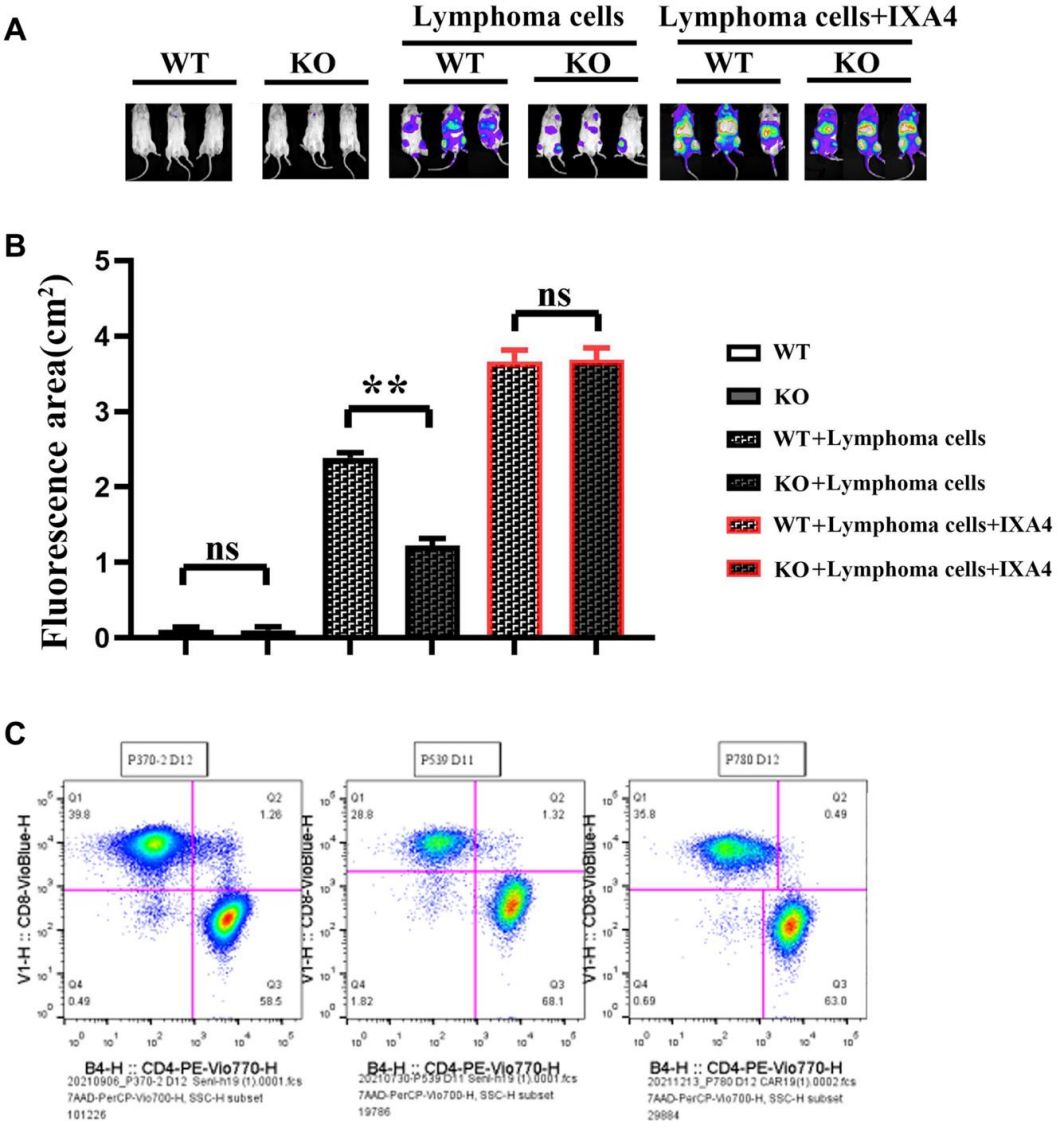
**Imaging of the effect of mouse tumor transplantation and myeloid-specific knockout Notch-1 on lymphoma progression and scale plot of CD4+/CD8+.** (**A**) Results of mouse tumor transplantation and imaging; (**B**) Fluorescence area in each group; (**C**) Ratio diagram of CD4+/CD8+. (WT group vs. KO group; WT + lymphoma cell group vs. KO + lymphoma cell group; WT + lymphoma cell + IXA4 group vs. KO + lymphoma cell + IXA4 group; ^**^P < 0.01, ^ns^*P* > 0.05; *N* = 3).

Lymphoma models were established by Raji-Luc-GFP cell injection into immunodeficient mice. The results showed that there was no fluorescence in nude mice in WT group and KO group. The fluorescence area significantly decreased in KO + lymphoma cell group compared with WT +lymphoma cell group, while it significantly increased in KO + lymphoma cell + IXA4 group compared with WT + lymphoma cell + IXA4 group, without significant differences (Figure 6).

### Knockout of Notch-1 could promote proliferation of CAR-T cells

**Figure 7:**
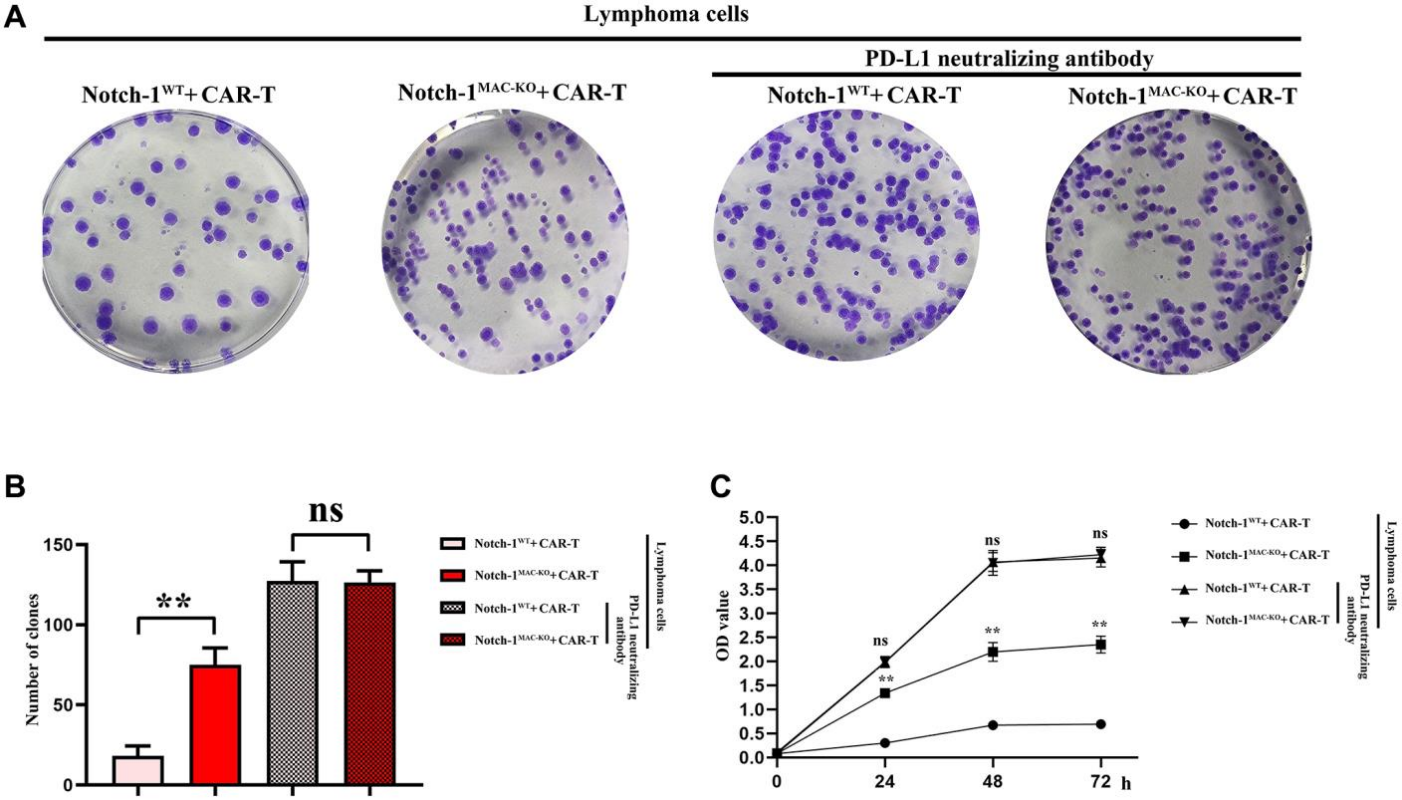
**CAR-T cell proliferation assay.** (**A**) Colony formation assay; (**B**) Data statistics of number of colonies; (**C**) CCK8 assay. (WT + lymphoma cell + CAR-T group vs. KO + lymphoma cell + CAR-T group; WT + lymphoma cell + CAR-T + PD-L1 neutralizing antibody group vs. KO + lymphoma cell + CAR-T + PD-L1 neutralizing antibody group; ^**^P < 0.01, ^ns^*P* > 0.05; *N* = 3).

To further explore the effect of Notch-1 knockout on CAR-T cells, colony formation assay and CCK8 assay were performed. The results showed that the cell proliferation capacity and the number of colonies were significantly inhibited in WT + lymphoma cell + CAR-T group, but they were significantly improved in KO + lymphoma cell + CAR-T group. PD-L1 neutralizing antibody significantly restored the cell proliferation capacity (Figure 7). The findings proved that the activation of the Notch-1 signaling pathway can suppress the CAR-T cell proliferation, and Notch-1 knockout or PD-L1 neutralizing antibody can significantly restore the proliferation capacity of CAR-T cells.

### Knockout of Notch-1 could reduce apoptosis of CAR-T cells

**Figure 8:**
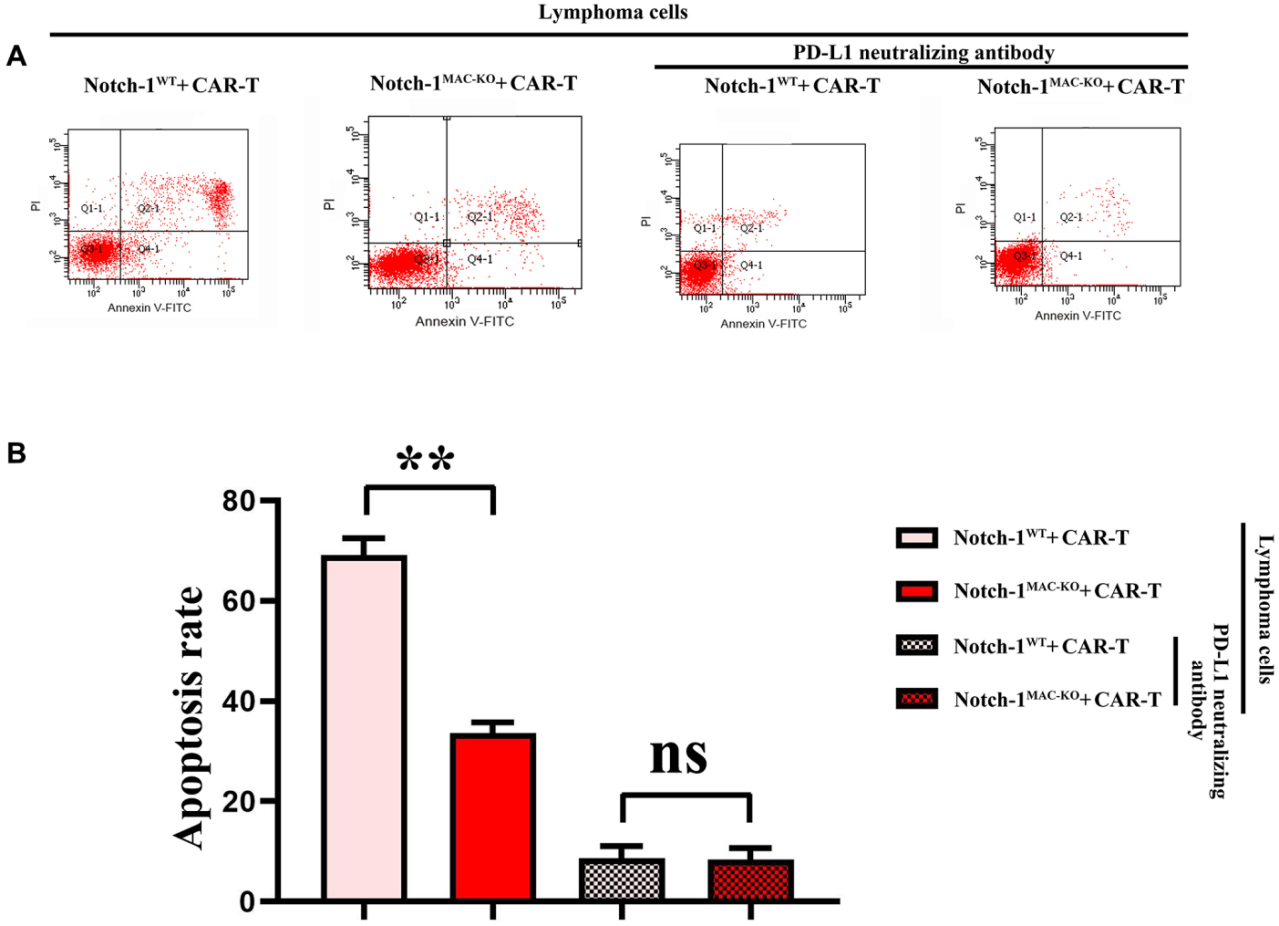
**Flow cytometry of CAR-T cell apoptosis.** (**A**) CAR-T cell apoptosis; (**B**) Data statistics of number of apoptotic CAR-T cells. (WT + lymphoma cell + CAR-T group vs. KO + lymphoma cell + CAR-T group; WT + lymphoma cell + CAR-T + PD-L1 neutralizing antibody group vs. KO + lymphoma cell + CAR-T + PD-L1 neutralizing antibody group; ^**^P < 0.01, ^ns^*P* > 0.05; *N* = 3).

Furthermore, the effect of Notch-1 knockout on apoptosis of CAR-T cells was detected using flow cytometry. The results showed that the number of apoptotic cells increased significantly in WT + lymphoma cell + CAR-T group, and decreased significantly in KO + lymphoma cell + CAR-T group. After the PD-L1 neutralizing antibody was used, the level of apoptosis reached the bottom (Figure 8). The results demonstrated that the activation of the Notch-1 signaling pathway in macrophages can enhance the apoptosis of CAR-T cells, and Notch-1 knockout or PD-L1 neutralizing antibody can significantly inhibit the apoptosis of CAR-T cells. To sum up, all the above findings indicate that lymphoma cells activate the Notch-1/IRE1/XBP1 signaling pathway in macrophages and promote the secretion of IL-6, IL-4 and PD-L1, thus inhibiting the activity and proliferation of CAR-T cells and promoting their apoptosis.

## DISCUSSION

In this study, it was found that the activation of the Notch-1/IRE1/XBP1s signaling pathway in bone marrow-derived macrophages could promote the secretion of IL-6 and IL-4, further affecting the expression of PD-L1 in DLBCL. Then the role and potential regulatory mechanisms of this signaling pathway in bone marrow-derived macrophages were discussed.

The notch-1 signaling pathway is ubiquitous in various types of cells and has important effects on many biological processes such as cell growth, differentiation and immune regulation. Activating the Notch-1 pathway promotes the M1 polarization of macrophages, which in turn produces a variety of pro-inflammatory cytokines (e.g., IL-6, TNF-α, etc.,) [18, 19]. The Notch-1 signaling pathway also plays an important role in regulating the polarization of bone marrow-derived macrophages, and can regulate ERS. ERS is a self-protective mechanism of cells in response to ER dysfunction. IRE1/XBP1s signaling pathway, as the core pathway of ERS, is crucial in regulating cell survival, apoptosis and inflammatory response [20]. In macrophages, the IRE1/XBP1s signaling pathway can regulate the inflammatory and immune responses. It has been found that activation of the IRE1/XBP1s signaling pathway can facilitate the production of IL-6, Il-1β and other inflammatory factors in macrophages, thus promoting the inflammatory response [21]. In addition, the IRE1/XBP1s signaling pathway also has important regulatory functions in macrophages in the tumor microenvironment, such as modulating tumor-associated macrophage (TAM) polarization and anti-tumor immunity [22]. In this study, activation of the Notch-1/IRE1/XBP1s signaling pathway in macrophages might further affect the development of DLBCL by regulating the secretion of IL-6 and IL-4, suggesting that the Notch-1/IRE1/XBP1s signaling pathway may influence the secretion of IL-6 and IL-4 by regulating the inflammatory/immune cell balance. Activation of the Notch-1 signaling pathway can enhance Th1 type immune response, thus promoting the secretion of IL-6 [23, 24], and activation of the IRE1/XBP1s signaling pathway can regulate the secretion of IL-1β, IL-6 and TNF-α [25].

PD-L1 is an important immune checkpoint molecule, which is highly expressed on the surface of tumor cells and can bind to its receptor PD-1 to inhibit the activation, proliferation and cytotoxicity of T cells, causing tumor immune escape [26]. In DLBCL, a high expression of PD-L1 is usually associated with poor prognosis [27]. The mechanism of tumor immune escape caused by PD-L1 in DLBCL involves different signaling pathways and regulatory factors. Bone marrow-derived macrophages play an important role in the tumor microenvironment by secreting various cytokines, such as IL-6 and IL-4, thereby regulating the growth, invasion and migration of tumor cells. In this study, activation of the Notch-1/IRE1/XBP1s signaling pathway in bone marrow-derived macrophages could enhance the secretion of IL-6 and IL-4, further increasing the expression of PD-L1 in DLBCL.

CAR-T cell therapy is a revolutionary immunotherapy, in which autologous T cells are genetically engineered to recognize and attack specific tumor cells. However, the application of CAR-T cell therapy in DLBCL is influenced by the PD-L1-mediated immune escape [28]. PD-L1 can induce CAR-T cell death, which reduces the efficacy of CAR-T cell therapy. Therefore, combining anti-PD-L1 antibodies in CAR-T cell therapy may be a promising strategy to improve the efficacy of CAR-T cell therapy. In this study, the role and mechanism of PD-L1 in tumor immune escape were explored, with particular focus on the promotion of IL-6 and IL-4 secretion by activation of the Notch-1/IRE1/XBP1s signaling pathway in macrophages. In the future, we need to further explore how to effectively leverage these mechanisms to optimize the CAR-T cell therapy, and to develop novel therapeutic strategies targeting PD-L1.

In conclusion, the role of the Notch-1/IRE1/XBP1s signaling pathway in bone marrow-derived macrophages in DLBCL and its potential mechanism were revealed to some extent in this study, which provide important clues for further study. However, more experiments should be conducted to fully understand the specific mechanisms of the Notch-1/IRE1/XBP1s signaling pathway in tumor development and immune escape. In the future, *in vivo* experiments and in-depth research on other signaling pathways and the interaction between cytokines can be performed, which are expected to provide new strategies and targets for the treatment of malignancies such as DLBCL, thus producing better therapeutic effects.
